# Cataract Surgery in Pseudoexfoliation Syndrome Using the Eight-Chop Technique

**DOI:** 10.3390/jpm15090396

**Published:** 2025-08-25

**Authors:** Tsuyoshi Sato

**Affiliations:** Department of Ophthalmology, Sato Eye Clinic, Nemoto 3-3, Matsudo-shi 271-0077, Chiba-ken, Japan; perfect-eightchop@sato-ganka.com

**Keywords:** cataract surgery, corneal endothelial cell, eight-chop technique, personalized treatment, phacoemulsification, pseudoexfoliation syndrome

## Abstract

**Objectives:** This study aimed to evaluate the safety and efficacy of the eight-chop technique in cataract surgery in patients with pseudoexfoliation (PEX) syndrome and assess the intraoperative parameters, changes in corneal endothelial cells, intraocular pressure (IOP), and intraoperative complications. **Methods:** This technique was applied in patients with and without PEX syndrome. Preoperative and postoperative assessments were conducted on best-corrected visual acuity, IOP, corneal endothelial cell density (CECD), coefficient of variation, percentage of hexagonal cells, and central corneal thickness. Intraoperative recordings included operative time, phaco time, aspiration time, cumulative dissipated energy (CDE), and fluid of volume used. **Results:** We analyzed 150 eyes from 150 patients (mean age, 75.5 ± 5.7 years; 59 men, 91 women). In the PEX group, operative time, phaco time, aspiration time, CDE, and volume of fluid used were 6.7 min, 17.4 s, 85.2 s, 6.91 µJ, and 33.4 mL, respectively, demonstrating favorable surgical metrics. On the other hand, in the control group, operative time, phaco time, aspiration time, CDE, and volume of fluid used were 4.5 min, 14.3 s, 64.0 s, 5.83 µJ, and 25.5 mL, respectively. In addition, CECD losses were 3.7% at week 7 and 2.7% at week 19 in the PEX group and 2.7% and 1.6%, respectively, in the control group. Significant decreases were observed at 7 and 19 weeks postoperatively in the PEX and control groups. No eye in the PEX group required a capsular tension ring due to zonular dialysis. **Conclusions:** The eight-chop technique in cataract surgery demonstrates excellent intraoperative parameters in patients with PEX, is effective against zonular weakness, and does not require the use of a capsular tension ring. This technique will aid in establishing personalized treatment strategies and improve cataract management and treatment.

## 1. Introduction

Pseudoexfoliation (PEX) syndrome is an age-related fibrillopathy characterized by the accumulation and deposition of white fibrillar exfoliation material in ocular and extraocular tissue [1,2,3]. Pseudoexfoliative materials comprise various extracellular matrix components such as fibrillin-1, elastin, fibronectin, and cross-linked glycoproteins [4,5,6]. PEX deposits are located extracellularly around blood vessels and in the connective tissues around the heart, lungs, and brain [7,8,9]. It has been definitively linked to Alzheimer’s disease, cerebral atrophy, ischemic heart disease, renal artery stenosis, aortic aneurysms, and systemic hypertension, among other conditions [1,7]. PEX has been detected on the ocular surface and in the anterior segment of the eye [1,2,7].

PEX frequently causes PEX glaucoma, accounting for 25–70% of glaucoma cases worldwide, making it the primary cause of open-angle glaucoma [2]. Other studies have demonstrated that patients with PEX have lower corneal endothelial cell density (CECD) [10,11,12] and their pupils are 21% smaller than those of healthy individuals [13]. Additionally, in patients with PEX, pseudoexfoliative deposits form in the zonules and zonular lamellae. This leads to zonular weakness and lens subluxation [1,14]. Furthermore, high incidences of intraoperative floppy iris syndrome and zonular rupture have been reported [15]. In a large-scale study, patients with PEX had a 2.68-fold higher risk of developing complications during cataract surgery; zonular weakness in PEX may lead to complications, such as zonular rupture, vitreous loss, and posterior capsule rupture [16,17].

It has been reported that the eight-chop technique for cataract surgery involves reduced operative and phaco time, less cumulative dissipated energy (CDE), reduced aspiration time, and less volume of fluid used than other surgical methods such as divide-and-conquer, phaco-chop. In addition, postoperative corneal endothelial cell loss is extremely low, and postoperative intraocular pressure (IOP) decreases [18,19,20,21,22]. Furthermore, even in cases with poor pupil dilation or a shallow anterior chamber depth, the postoperative reduction in corneal endothelial cell density was superior to that of conventional surgical techniques, IOP reduction was maintained, and the incidence of intraoperative complications was extremely low [19,22]. Therefore, the eight-chop technique may be effective in PEX cases with a high risk of intraoperative complications during cataract surgery due to the possibility of corneal endothelial cell loss and IOP elevation.

Modern phaco machines can record various critical parameters during cataract surgery, including CDE, phaco time, aspiration time, volume of fluid used, and duration of the surgical procedure. Limited studies have analyzed intraoperative parameters in patients with PEX syndrome undergoing phacoemulsification surgery [17]. Therefore, we aimed to evaluate the efficacy of the eight-chop technique in patients with PEX syndrome who underwent cataract surgery. We examined intraoperative parameters, changes in corneal endothelial cells and IOP, as well as intraoperative complications. Furthermore, we will verify the effectiveness of the eight-chop technique in patients with PEX syndrome, who are highly susceptible to corneal endothelial cell vulnerability and intraoperative complications. Additionally, we conducted an extensive literature search to examine the superiority of the eight-chop technique. We focused particularly on intraoperative parameters and postoperative corneal endothelial cell density loss. Our research on the eight-chop technique for patients with PEX syndrome aims to promote the development of personalized treatment strategies and ultimately contribute to cataract surgery.

## 2. Materials and Methods

### 2.1. Ethical Considerations

The institutional review board reviewed and provided its approval for the study protocol to be used. This approval was granted with adherence to the tenets of the Declaration of Helsinki. After carefully articulating the study’s nature and potential outcomes, consent to participate was obtained for each patient (approval number 20140901).

### 2.2. Study Population

The study focused on patients with cataracts who underwent phacoemulsification and posterior chamber intraocular lens (IOL) implantation between November 2014 and July 2024. Subsequently, these patients visited the Sato Eye Clinic in Matsudo City, Chiba Prefecture, Japan. The presence of PEX materials in the anterior segment was determined by observing fibrillary deposits on the pupillary margin, anterior lens capsule, or both, using a slit lamp microscope (SM-70N; TAKAGI, Tokyo, Japan). We excluded patients with corneal disease or opacity, uveitis, diabetic retinopathy, white cataracts, preoperative CECD < 2000 cells/mm^2^, and a history of ocular trauma or surgery.

### 2.3. Preoperative Assessment

All patients were thoroughly evaluated using a slit-lamp and retinal biomicroscopy (DRI OCT; Topcon Corporation, Tokyo, Japan). Best corrected visual acuity (BCVA) and IOP were examined preoperatively. CECD (cells/mm^2^), central corneal thickness (CCT), coefficient of variation (CV), and percentage of hexagonal cells (PHC) were assessed using a non-contact specular microscope (EM-3000; Topcon Corporation, Tokyo, Japan). The hardness of the nucleus was determined through the utilization of the Emery classification [23]. Axial length and anterior chamber depth were evaluated utilizing a swept-source optical coherence tomograph with a laser wavelength of 1060 nm (OA-2000; TOMEY, Tokyo, Japan) [24].

### 2.4. Surgical Technique

All patients were managed by the same surgeon, who has experience with the eight-chop technique using the phacoemulsification system (Centurion^®^; Alcon Laboratories, Inc., Irvine, CA, USA). The surgical instruments used during the study remained the same throughout. In each case, a 3.0 mm steel keratome (MSL3; MANI, Inc., Tochigi, Japan) was used to create a temporal clear corneal incision. The next step in the procedure was to administer sodium hyaluronate through injection into the anterior chamber. This was followed by the creation of a 6.2–6.5 mm continuous curvilinear capsulorrhexis using capsule forceps (F-2055; M.E.Technica, Tokyo, Japan). Hydrodissection was successfully completed using a 27-G cannula (AMO Japan, Inc., Tokyo, Japan). The lens nucleus was divided into eight sections using the eight-chop technique. Eight-chopper I or II was used for division depending on the lens hardness which was grade II or III. At the plane of the iris, the eight segments were delicately phacoemulsified and aspirated. Subsequently, the capsular bag was delicately extracted with an irrigation/aspiration tip to eliminate the cortical materials. The ophthalmic viscosurgical device was applied, and a foldable three-piece IOL (Acrysof^®^ MN60AC; Alcon Laboratories, Inc., Fort Worth, TX, USA) with polymethyl methacrylate haptics was gently inserted into the capsular bag through a precise injection process. The next step was to delicately remove the ophthalmic viscosurgical device. Postoperative treatment involved replacing the anterior chamber with a balanced salt solution containing moxifloxacin (0.5 mg/mL).

Intraoperative outcome measures included operative time in minutes, phaco time in seconds, aspiration time in seconds, CDE in microjoule, volume of fluid used in milliliters, and incidence of intraoperative complications. The operative time was carefully measured from the start of the corneal incision to the conclusion of the aspiration of the ophthalmic viscosurgical device. All surgeries were documented utilizing a camera (MKC-704KHD; Ikegami Tsushinki Co., Ltd., Tokyo, Japan), and video images were preserved on a hard disk.

### 2.5. Intraoperative Signs of Zonular Weakness

Zonular weakness was diagnosed if two or more of the following findings are observed during surgery: prominent striated changes in the anterior capsule during continuous curvilinear capsulorrhexis preparation, eccentric displacement of the lens nucleus during phacoemulsification, difficulty in rotating the lens nucleus, striated changes in the posterior capsule, and difficulty in aspiration during cortical removal [25].

### 2.6. Literature Review of the Eight-Chop Technique and Other Surgical Techniques

We searched for papers on phacoemulsification cataract surgery over the past seven years. Papers were selected if they clearly described the surgical technique and included at least two of the following items: operative time, phaco time, aspiration time, CDE, volume of fluid used, and CECD loss. For the CECD loss, data from three weeks to six months postoperatively were used, and one paper in which the cell density increased postoperatively was excluded.

### 2.7. Data Collection and Statistical Analysis

Patients were closely monitored on postoperative days 1 and 2 and weeks 1, 3, 7, and 19. Postoperative outcome assessments including BCVA, IOP, CCT, CV, PHC, and CECD were conducted at 7 and 19 weeks postoperatively.

Statistical analyses were performed using the Mann–Whitney U test to compare the results obtained from the two groups. The pre- and postoperative BCVA, IOP, CV, PHC, CCT, and CECD values were compared using a paired t-test. The chi-square test was used to determine whether sex-related differences were observed between the PEX and control groups. The sample size was determined using G-Power software (version 3.1.9.7) [26] to ensure that the study had sufficient statistical power to detect significant differences in the results. The parameters for the calculation were based on the data in our paper [18]. A *p*-value < 0.05 was considered statistically significant.

## 3. Results

### 3.1. Characteristics of the Participants

In this study, we carefully examined 150 eyes of 150 patients with cataract who were treated with phacoemulsification and IOL implantation. Table 1 presents patient characteristics and intraoperative parameters. There were no significant differences between the PEX and control groups in regard to mean age, sex distribution, anterior chamber depth, and lens hardness (*p* = 0.55, *p* = 0.62, *p* = 0.97, and *p* = 0.66, respectively). However, the two groups differed significantly in terms of preoperative pupil size, operative time, phaco time, aspiration time, CDE, and volume of fluid used (*p* < 0.01, *p* < 0.01, *p* = 0.02, *p* < 0.01, *p* = 0.04, and *p* < 0.01, respectively). Glaucoma was detected in 21 eyes in the PEX group.

### 3.2. Changes in CECD

Table 2 shows the pre- and postoperative changes in CECD. CECD differed significantly between the PEX and control groups preoperatively and at 7 and 19 weeks postoperatively (all *p* < 0.01). In the PEX and control groups, significant decreases were observed at 7 and 19 weeks postoperatively (*p* < 0.01 or *p* = 0.01). Regarding the percentage decrease in CECD, no significant differences were observed between the two groups at 7 and 19 weeks postoperatively (*p* = 0.87 and *p* = 0.29, respectively).

### 3.3. Changes in CCT, CV, and PHC

Table 3 shows the pre- and postoperative changes in CCT, CV, and PHC. No significant differences were observed in CCT preoperatively and at 7 and 19 weeks postoperatively between the PEX and control groups (*p* = 0.60, *p* = 0.59, and *p* = 0.82, respectively). Significant differences were observed in CV at 7 weeks postoperatively (*p* < 0.01); however, no significant differences were observed preoperatively and 19 weeks postoperatively (*p* = 0.51 and *p* = 0.06, respectively) between the PEX and control groups. Significant differences were observed in PHC between the PEX and control groups at 7 and 19 weeks postoperatively (all *p* < 0.01); however, no significant differences were observed between the two groups preoperatively (*p* = 0.70).

### 3.4. Changes in IOP

Table 4 shows the changes in IOP. No significant differences were observed in the IOP preoperatively and at 7 and 19 weeks postoperatively between the PEX and control groups (*p* = 0.34, *p* = 0.54, and *p* = 0.53, respectively). In the PEX and control groups, significant decreases were observed at 7 and 19 weeks postoperatively (all *p* < 0.01). Regarding the percentage decrease in IOP, no significant differences were observed between the two groups at 7 and 19 weeks postoperatively (*p* = 0.89 and *p* = 0.28, respectively).

### 3.5. Changes in BCVA over Time

Table 5 shows the changes in the BCVA. No significant differences were observed in BCVA preoperatively and at 7 and 19 weeks postoperatively between the PEX and control groups (*p* = 0.86, *p* = 0.08, and *p* = 0.15, respectively). In the PEX and control groups, BCVA differed significantly between the preoperative period and both at 7 and 19 weeks postoperatively (all *p* < 0.01).

### 3.6. Intraoperative Parameters and Corneal Endothelial Cell Loss

Table 6 provides a summary of intraoperative parameters and corneal endothelial cell density loss according various phacoemulsification cataract surgery techniques.

### 3.7. Complications and Additional Procedures During Surgery

No intraoperative complications or capsulorrhexis tears were observed in the PEX and control groups. During surgery, 13 (17.3%) eyes exhibited zonular weakness, but no cases required a CTR (capsular tension ring). We examined intraoperative video recordings at the end of surgery to confirm the presence or absence of IOL decentration and zonular dialysis in 13 cases with zonular weakness. The IOL was fixed in the central cornea in all cases, and no zonular dialysis was observed. In the PEX group, 12 (16.0%) patients required iris hooks because of inadequate pupil dilation.

## 4. Discussion

In this study, the operative times for the PEX and control groups were 6.7 and 4.5 min, respectively. In the PEX group, operative time for cases using iris hooks included approximately 3.3 min required [19] for placing iris hooks, which was one of the causes of the prolonged operative time in the PEX group. Reportedly, other surgical procedures take between 7.6 and 19.6 min [38,39], demonstrating that the eight-chop technique shortens the operative time. Furthermore, the phaco and aspiration times were minimal, and the CDE was low. The volume of fluid used in other studies ranged from 46 to 139 mL [17,39,40,41]; however, in this study, 33.4 and 25.5 mL were used in the PEX and control groups, respectively. The eight-chop technique involves mechanically dividing the nucleus into eight pieces before phacoemulsification. This decreases the amount of ultrasonic energy used and allows for more efficient removal of fragmented nuclei, resulting in shorter phaco and aspiration times and a reduction in CDE [18,19,20,21,22]. The eight-chop technique is comparable to femtosecond laser-assisted cataract surgery because it divides the lens nucleus without using ultrasonic energy.

Statistically significant differences were observed in phaco time, aspiration time, and volume of fluid used between patients with and without PEX [42,43]. In the PEX group, zonule weakness and insufficient mydriasis created the need for more careful surgery [42,43]. There were significant differences between the PEX and control groups in terms of operative time, phaco time, aspiration time, CDE, and the volume of fluid used. In the PEX group, 13 patients exhibited zonular weakness. In these cases, rotation of the lens nucleus after hydrodissection was difficult, and some eccentric displacement occurred when the Eight-chopper was inserted into the lens nucleus, requiring careful manipulation to avoid occurring zonular dialysis. In addition, the posterior lens capsule appeared to be aspirated during lens cortex removal, which may have resulted in a longer procedure and an increase in the volume of fluid used. Furthermore, it appears that division of the lens nucleus and phacoemulsification required additional time because of poor mydriasis.

Research has shown that CECD decreases in PEX eyes compared to non-PEX eyes [39]. In this study, we confirmed that the PEX group showed a significant decrease in preoperative CECD compared to the control group. The reduction rates of CECD after cataract surgery were 9.0–11.4% and 3.4–8.1% in PEX eyes and non-PEX eyes, respectively [39,41], demonstrating a higher rate in PEX eyes. In the PEX group, this study showed a decrease of 3.7% and 2.7% at 7 and 19 weeks postoperatively, respectively. The control group showed a decrease of 2.7% and 1.6% at 7 and 19 weeks postoperatively, respectively, demonstrating a higher rate in PEX eyes. Furthermore, the reduction rates in both groups were extremely low compared with those reported in previous studies. The minimally invasive nature of the eight-chop technique may have suppressed the decrease in CECD due to the fragility of the corneal endothelial cells in PEX eyes. The eight-chop technique is relatively noninvasive, which may be attributed to the minimal ultrasonic energy required and the low volume of fluid used. The flow of fluid during surgery results in reduced loss or damage to corneal endothelial cells. This is consistent with the surgical results reported to date [18,19,20,21,22].

The anterior chamber, where the lens is removed, has limited space, making corneal endothelial cells susceptible to damage from ultrasound energy during surgery [44,45,46]. Thus, a decrease in CECD during phacoemulsification is unavoidable. Despite the advantages of phacoemulsification, postoperative CECD reduction remains a significant concern. In addition, assessing CECD reduction helps to compare surgical techniques because it reflects intraoperative damage to the intraocular tissues [36]. Our research suggests that the eight-chop technique might have the potential to reduce the surgical invasion of intraocular tissues, including the corneal endothelium, ciliary body, and Schlemm’s canal. Considering that modern cataract surgery aims to improve vision and minimize damage to corneal endothelial cells in patients with cataracts complicated by other eye diseases, the eight-chop technique is a very useful surgical approach.

CCT is a clear indicator of corneal endothelial function [27,47]. Previous studies have reported that the rate of CCT increase in PEX eyes was higher than that in non-PEX eyes at 4 weeks postoperatively, but no significant difference was observed at 12 weeks postoperatively [39]. However, we found no significant difference in CCT between the PEX group and control groups before and after surgery. CCT differed significantly between the preoperative period and at 7 weeks postoperatively in the PEX group, whereas no significant difference was observed in the control group. This result indicates that corneal endothelial cell function deteriorates in the PEX group at 7 weeks postoperatively, aligning with the findings reported by Hayashi et al. [39]. CV indicates the uniformity of endothelial cell size, whereas PHC indicates variability in hexagonal cell shape, and both are hallmarks of healing responses after injury [47]. Previous studies have reported a tendency toward decreased PHC and increased CV in PEX eyes [48], whereas other reports have found no significant differences in PHC and CV between preoperative and postoperative measurements in cataract surgery [41]. This study definitively shows no significant differences in CV and PHC between the PEX and control groups preoperatively. However, significant differences in CV and PHC were observed at 7 weeks postoperatively. Additionally significant differences in PHC were observed at 19 weeks postoperatively. These results indicate that the endothelial repair and healing mechanisms may be impaired in the PEX group postoperatively.

Preoperative IOP was significantly higher in PEX eyes compared to non-PEX eyes [40,49], and IOP decreased by 10.5–30.6% and 2.3–3.9% in PEX and non-PEX eyes postoperatively, respectively [40,49]. Preoperative IOP predicts postoperative IOP reduction [50,51]. In this study, the preoperative IOP was not significantly higher in the PEX group than in the control group. Postoperative IOP reduction rates were 12.9% and 10.0%, respectively, at 19 weeks postoperatively. In this study, both groups showed a high rate of IOP reduction of at least 10%. This is likely because the eight-chop technique uses a small amount of fluid, which reduces the impact on the trabecular meshwork cells and Schlemm’s canal cells. This maintains the normal function of the trabecular meshwork, resulting in a greater reduction in IOP.

The incidence of complications during PEX eye surgery is high, and the main complications include zonular instability, vitreous loss, poor mydriasis, and lens subluxation [16,17,52,53,54]. In PEX eyes, the frequency of using a CTR due to zonular weakness was 3.96%, and the frequency of posterior capsule rupture was 5.40%, which are reported to be 30 and 13 times higher than those in non-PEX eyes, respectively [17]. In this study, zonular weakness was observed in 13 out of 75 PEX eyes, but no eyes required CTR due to zonular dialysis. Furthermore, there was no IOL eccentricity or zonular dialysis at the end of the surgery. With the eight-chop technique, the combined use of a nucleus sustainer and Lance-chopper allows phacoemulsification without stress on the zonules or posterior capsule, even in cases with a hard lens [18,20]. In the eight-chop technique, an Eight-chopper with a sharp tip is inserted into the lens nucleus and divided without striking the lens nucleus with an ultrasound tip, so only a small amount of force is required to push the lens nucleus. If zonule weakness is detected when inserting the Eight-chopper into the lens nucleus, zonular dialysis is prevented by using a nucleus sustainer along with the Lance-chopper. If the lens nucleus is divided into eight segments, phacoemulsification of the lens nucleus can be performed without a CTR. At this stage of the procedure, the ultrasonic tip is not inserted into the lens nucleus to avoid stress on the zonules or posterior lens capsule. In cases of zonule weakness alone within the continuous curvilinear capsulorrhexis, inserting a CTR did not improve the accuracy of refractive prediction [55]. Therefore, CTR should be used with caution. A shorter interval between cataract surgery and in-the-bag dislocation has been reported in eyes where a CTR was inserted [56,57,58]. The usefulness of CTR insertion for preventing late IOL dislocations remains unclear [59]. In addition, this study revealed that inserting a three-piece IOL into a capsular bag could be an effective strategy for preventing capsular bag eccentricity.

The prechop technique is the best way to reduce the amount of ultrasonic energy and surgical trauma to intraocular tissues. It does this by mechanically dividing the lens into four segments before phacoemulsification [60]. This feature shares many similarities with the benefits of femtosecond laser-assisted cataract surgery. However, from a medical and economic perspective, it is potentially superior to femtosecond laser-assisted cataract surgery. Despite the numerous noteworthy features of the prechop technique, it is not widely adopted as a surgical technique because of the inherent challenges associated with operating the prechopper [18,61]. Hence, an enhanced version of the pre-chop technique, known as the eight-chop technique, has been shown to improve operational efficiency [18].

As shown in Table 6, the average operative time for the eight-chop technique was 3.7 to 4.6 min for the Grade II and III groups [18,19]. This is significantly shorter than the 12.3 to 19.2 min reported for other techniques [32,37,38]. The eight-chop technique can be completed in less than 10 min, even in cases with hard lens nuclei [20]. This is reportedly shorter than the 12 min it takes for the phaco-chop technique [37]. Using the eight-chop technique, the phaco time was reported to be 11.6 s [18]. In contrast, other surgical techniques reported phaco time ranging from 29.5 to 122.4 s [28,29,30,31,32,33,38]. Therefore, the eight-chop technique reduces phaco time. Furthermore, the volume of fluid used in this technique is 22.9 mL, which is significantly less than 32.8 to 354.7 mL reported in other techniques [18,28,30,35,36,38]. Corneal endothelial cell density is the true summation of intraocular impact during surgery, and its assessment is essential for comparing various techniques [36]. A 4.3–31.8% decrease in corneal endothelial cell density after cataract surgery in the first few postoperative months has been reported in previous studies [27,29,30,31,32,34,36,37,38,62]; however, using the eight-chop technique, the decrease was 0.9%, 1.0%, and 5.3% in the Grade II, III, and IV groups, respectively [18]. After careful consideration of the available data, it seems that the eight-chop technique has the potential to be a surgical technique that is both highly efficient and less invasive than other surgical techniques.

This study has some limitations. First, the surgical outcomes were not directly compared with the prechop technique, phaco-chop technique, or divide-and-conquer technique. When evaluating the current results, it would be beneficial to carefully consider this point. In the future, it would be beneficial to directly compare the surgical results of the phaco-chop or divide-and-conquer technique with those of the eight-chop technique for more accurate verification. However, numerous other studies using the phaco-chop technique or the divide-and-conquer technique have been conducted, and we are confident that our results can be evaluated by comparison with these studies. Second, we did not evaluate surgical outcomes based on the severity of PEX and zonular weakness. Further evaluation is necessary in the future to consider the severity of these conditions. Third, the observation period was short (19 weeks postoperatively); therefore, the incidences of pseudophakodonesis, anterior capsule contraction, and IOL decentration could not be evaluated. Fourth, since the purpose of this paper was to examine early postoperative outcomes, long-term follow-up is currently unavailable, and further verification is necessary. Fifth, considering intraoperative complications, we always use 3-piece IOLs in both groups. Therefore, complications associated with the use of 1-piece IOLs are unknown.

## 5. Conclusions

The eight-chop technique demonstrated excellent intraoperative parameters for cataracts with PEX, with only a slight decrease in CECD and a reduction in IOP. Other studies have reported a high incidence of intraoperative complications, such as zonular dehiscence and posterior capsule rupture. However, the present study used the eight-chop technique and no complications were reported. It is thought that the eight-chop technique is effective in patients with PEX syndrome, who are characterized by corneal endothelial cell fragility and a high incidence of intraoperative complications. The eight-chop technique is effective for zonular weakness and does not require a CTR. Research on the eight-chop technique for cataracts with PEX will establish personalized treatment strategies and improve cataract management and treatment.

## Figures and Tables

**Table 1:** Preoperative characteristics and intraoperative parameters.

Characteristic/Parameter	PEX Group	Control Group	*p*-Value
Number of eyes	75	75	
Age (y)	75.4 ± 7.3	75.7 ± 3.5	0.55 ^a^
Gender: Men	28 (37.3%)	31 (41.3%)	0.62 ^b^
Women	47 (62.7%)	44 (58.7%)	
Glaucoma	21 (28.0%)	0	
Anterior chamber depth (mm)	3.27 ± 0.57	3.27 ± 0.36	0.97 ^a^
Preoperative pupil size (mm)	6.2 ± 1.0	6.9 ± 0.5	<0.01 ^c^
Lens hardness	2.3 ± 0.3	2.3 ± 0.3	0.66 ^a^
Operative time (min)	6.7 ± 3.4	4.5 ± 0.8	<0.01 ^c^
Phaco time (s)	17.4 ± 7.8	14.3 ± 3.9	0.02 ^c^
Aspiration time (s)	85.2 ± 26.6	64.0 ± 12.5	<0.01 ^c^
CDE (µJ)	6.91 ± 2.87	5.83 ± 1.57	0.04 ^c^
Volume of fluid used (mL)	33.4 ± 10.9	25.5 ± 5.4	<0.01 ^c^

Values are expressed as mean ± standard deviation or percentages, unless otherwise noted. ^a^ No significant differences were found between the groups using the Mann–Whitney U test. ^b^ No significant differences were found between the groups using the chi-square test. ^c^ Significant differences were found between the groups using the Mann–Whitney U test. PEX, pseudoexfoliation; CDE, cumulative dissipated energy.

**Table 2:** Pre- and postoperative CECD values.

	Mean CECD ± SD (% Decrease)		
Time Period	PEX Group (*n* = 59)	Control Group (*n* = 75)	*p*-Value
Preoperatively	2532 ± 342	2710 ± 248	<0.01 ^a^
7 weeks postoperatively	2432 ± 400 ^b^	2637 ± 245 ^b^	<0.01 ^a^
% Decrease	3.7 ± 12.7	2.7 ± 2.6	0.87 ^c^
19 weeks postoperatively	2461 ± 381 ^b^	2668 ± 256 ^b^	<0.01 ^a^
% Decrease	2.7 ± 8.8	1.6 ± 2.2	0.29 ^c^

Values are presented as mean ± standard deviation. ^a^ Significant differences were found between groups using the Mann–Whitney U test. ^b^ Significant differences were found between the preoperative and respective time values using a paired *t*-test. ^c^ No significant differences were found between the groups using the Mann–Whitney U test. CECD, corneal endothelial cell density; SD, standard deviation; PEX, pseudoexfoliation.

**Table 3:** Pre- and postoperative endothelial CCT, CV, and PHC.

Time Period	PEX Group (*n* = 54)	Control Group (*n* = 75)	*p*-Value
CCT	Mean ± SD		
Preoperatively	530 ± 34.4	534 ± 31.0	0.60 ^a^
7 weeks postoperatively	540 ± 40.5 ^c^	536 ± 29.8 ^d^	0.59 ^a^
19 weeks postoperatively	532 ± 36.5 ^d^	533 ± 29.9 ^d^	0.82 ^a^
CV	Mean ± SD		
Preoperatively	39.8 ± 6.3	39.2 ± 6.1	0.51 ^a^
7 weeks postoperatively	41.5 ± 5.6 ^d^	38.8 ± 5.5 ^d^	<0.01 ^b^
19 weeks postoperatively	39.1 ± 5.9 ^d^	37.1 ± 5.3 ^c^	0.06 ^a^
PHC	Mean ± SD		
Preoperatively	44.5 ± 7.0	45.3 ± 6.6	0.70 ^a^
7 weeks postoperatively	41.8 ± 7.0 ^c^	45.9 ± 6.4 ^d^	<0.01 ^b^
19 weeks postoperatively	44.5 ± 6.7 ^d^	47.9 ± 6.0 ^c^	0.01 ^b^

Values are presented as mean ± standard deviation. ^a^ No significant differences were found between the groups using the Mann–Whitney U test. ^b^ Significant differences were found between the groups using the Mann–Whitney U test. ^c^ Significant differences between the preoperative and respective time values using a paired *t*-test. ^d^ No significant differences were found between the preoperative and respective time values using a paired *t*-test. CCT, central corneal thickness; CV, coefficient of variation; PHC, percentage of hexagonal cells; PEX, pseudoexfoliation; SD, standard deviation.

**Table 4:** Mean IOP (mmHg) and mean decrease (%) in the IOP (mmHg) over time.

	Mean IOP ± SD (% Decrease)		
Time Period	PEX Group (*n* = 69)	Control Group (*n* = 75)	*p*-Value
Preoperatively	14.5 ± 3.0	14.0 ± 1.9	0.34 ^a^
7 weeks postoperatively	12.4 ± 3.1 ^b^	11.9 ± 1.5 ^b^	0.54 ^a^
% Decrease	13.4 ± 17.4	14.2 ± 9.2	0.89 ^a^
19 weeks postoperatively	12.4 ± 2.8 ^b^	12.5 ± 1.7 ^b^	0.53 ^a^
% Decrease	12.9 ± 16.1	10.0 ± 11.5	0.28 ^a^

Values are presented as mean ± standard deviation. ^a^ No significant differences were observed between the groups using the Mann–Whitney U test. ^b^ Significant differences were found between the preoperative and respective time values using a paired *t*-test. IOP, intraocular pressure; SD, standard deviation; PEX, pseudoexfoliation.

**Table 5:** Pre- and postoperative best-corrected visual acuity values.

	Best-Corrected Visual Acuity logMAR		
Time Period	PEX Group (*n* = 54)	Control Group (*n* = 75)	*p*-Value
Preoperatively	0.114 ± 0.211	0.087 ± 0.119	0.86 ^a^
7 weeks postoperatively	−0.059 ± 0.041 ^b^	−0.070 ± 0.026 ^b^	0.08 ^a^
19 weeks postoperatively	−0.062 ± 0.033 ^b^	−0.069 ± 0.027 ^b^	0.15 ^a^

Values represented as mean ± standard deviation. ^a^ No significant differences were found between the groups using the Mann–Whitney U test. ^b^ Significant differences were found between the groups using a paired *t*-test. logMAR, logarithmic minimum angle of resolution; PEX, pseudo-exfoliation.

**Table 6:** Results of intraoperative parameters and corneal endothelial cell loss by various phacoemulsification techniques.

Study	Year	Eyes	Surgical Technique	Operative-Time (min)	Phaco Time (s)	Aspiration-Time (s)	CDE (µJ)	VFU (mL)	CECD Loss (%)
Opala [27]	2025	80	Stop-and-chop	NR	NR	NR	4.19	NR	18.8
Spaulding [28]	2025	36	Stop-and-chop	NR	29.5	90.1	5.00	32.8	NR
Wang [29]	2025	123	Phaco-chop	NR	68.9	NR	18.2	NR	10.6
Wang [30]	2024	55	Phaco-chop	NR	30.6	NR	5.22	45.1	4.3
Fernández-Muñoz [31]	2023	30	Phaco-chop	NR	94.0	NR	20.11	NR	31.8
Eom [32]	2023	76	Phaco-chop	12.3	25.7	NR	NR	NR	8.1
Sinha [33]	2023	50	Stop-and-chop	NR	122.4	NR	6.9	NR	10.1
Tao [34]	2023	45	Reverse-chop	NR	NR	NR	7.53	NR	15.9
Cyril [35]	2022	82	Phaco-chop	NR	NR	NR	4.80	36.1	NR
Upadhyay [36]	2022	50	Crater-chop	NR	NR	NR	NR	105.9	4.4
Abdelmotaal [37]	2021	66	Phaco-chop	12.3	NR	NR	19.13	NR	15.2
Igarashi [38]	2019	32	Divide-and-conquer	19.2	45.9	NR	NR	354.7	8.5
Sato [19]	2024	65	Eight-chop	4.6	16.2	72.1	7.00	28.9	1.2
Sato [18]	2023	50	Eight-chop	3.7	11.6	NR	5.00	22.9	0.9
Present	2025	75	Eight-chop	6.7	17.4	85.2	6.91	33.4	2.7

CDE = cumulative dissipated energy. VFU = volume of fluid used. CECD loss = corneal endothelial cell density loss. NR = not reported.

## Data Availability

The data presented in this study are available upon request from the corresponding author due to privacy and ethical restrictions.

## Abbreviations

The following abbreviations are used in this manuscript:

PEX	Pseudoexfoliation
IOP	Intraocular pressure
CECD	Corneal endothelial cell density
CDE	Cumulative dissipated energy
IOL	Intraocular lens
BCVA	Best-corrected visual acuity
CCT	Central corneal thickness
CV	Coefficient of variation
PHC	Percentage of hexagonal cells
SD	Standard deviation
VFU	Volume of fluid used
NR	Not reported
CTR	Capsular tension ring
